# Influence of Mechanical Deformity on Joint Line Reconstruction

**DOI:** 10.3390/jcm14041264

**Published:** 2025-02-14

**Authors:** Anna Jungwirth-Weinberger, Maximilian F. Kasparek, Kirubakaran Pattabiraman, Arnab Sain, Maximilian Muellner, Tobias Scheidl, Oliver Haider, Thomas Muellner

**Affiliations:** 1 Department of Orthopedic Surgery and Traumatology, Evangelisches Krankenhaus, 1180 Vienna, Austriaarnabsain88@gmail.com (A.S.); tobias.scheidl@modul.at (T.S.);; 2 Vienna General Hospital, Medical University of Vienna, Waehringer Guertel 18–20, 1090 Vienna, Austria; 3 Department of Orthopedics, AIIMS, Sri Aurobindo Marg, Ansari Nagar, Ansari Nagar East, New Delhi 110029, India; 4 Center for Musculoskeletal Surgery, Charité—Universitätsmedizin Berlin, Klinik für Orthopädie, Schumannstraße 20, 10117 Berlin, Germany; muellner.max@gmail.com

**Keywords:** joint line restoration, joint line variability, total knee arthroplasty, varus knee, valgus knee

## Abstract

**Background**: Restoration of the joint line is important for an optimal outcome after total knee arthroplasty (TKA). The goal of this study was to analyze the accuracy of joint line reconstruction in conventionally performed TKA. The study evaluates the potential influences of mechanical deformity on joint line restoration. **Methods**: A total of 115 patients (58.3% female, mean age 72.4 years (52–89)) with 43 valgus and 72 varus knees were reviewed. A total of 36 patients underwent CR-TKA, and 79 underwent PS-TKA. The joint line was measured from the adductor tubercle to the joint line. **Results**: A total of 106 patients (92.2%) had the joint line restored within four millimeters. The distance increased significantly from preoperative (48.30 ± 6.35 mm) to postoperative 49.03 ± 6.29 mm (*p* = 0.003). Varus knees showed no significant change (*p* = 0.313), while valgus knees had a significant elevation (*p* = 0.0004). **Conclusions**: In conventional total knee arthroplasty (TKA), the distance from the adductor tubercle to the joint line slightly increased. However, in most patients, the joint line was restored within four millimeters. Valgus knees in particular are at risk for postoperative joint line elevation.

## 1. Introduction

Restoration of the joint line is important to restore the kinematics of the knee joint and is recommended to obtain the best function in primary TKA [1,2,3]. In most primary total knee arthroplasties, joint line restoration is achieved through a precise resection of the distal femur. This approach depends on preserving the bone of the distal femoral condyles to guide the bony resection and replacement. Bony landmarks can be utilized to estimate the native joint line position either by taking measurements from imaging of the contralateral knee or by using a fixed distance from a landmark [4].

Joint line elevation can occur from recutting the distal femur to improve the extension gap or from distal femoral bone loss in revision TKA. A systematic review by van Lieshout et al. showed that the variation between preoperative and postoperative joint lines should not exceed four millimeters, as elevation is associated with inferior clinical outcomes [2]. Cadaver studies have shown that elevation of the joint line can lead to mid-flexion laxity [5,6], and is associated with a reduced posterior condylar offset, patellar impingement, quadriceps weakness, anterior knee pain, and decreased range of motion [7,8]. Moreover, a joint line elevation might increase patellofemoral contact forces [9].

Recent studies show that neutral mechanical alignment resulted in a significantly greater deviation from the patient’s native joint line along the lateral compartment of the knee (lateral distal femoral condyle, lateral posterior femoral condyle, lateral tibial resection), compared to the restricted kinematic alignment technique [10]. Another study demonstrated that joint line modification in kinematic alignment improved functional activity but not patient satisfaction [11]. Additionally, robotic-assisted TKA restored joint line height and posterior offset more accurately compared to patients undergoing TKA with a conventional technique [12].

The influencing factors on joint line elevation during conventional TKA are still a matter of debate [3,13,14]. Therefore, the current study aims to analyze the accuracy of joint line reconstruction in conventionally performed TKA.

Moreover, the current study analyzes the potential influences of preoperative mechanical deformity on joint line restoration in conventionally performed TKA.

## 2. Materials and Methods

The current study examines a consecutive series of 115 patients undergoing unilateral primary TKA for osteoarthritis between 2017 and 2019 at a single institution. Patients with rheumatoid arthritis, previous surgery, posttraumatic deformities, or infection were excluded. Extraarticular deformities were excluded as well. The restoration of the joint line after TKA was retrospectively investigated on standardized anteroposterior pre- and postoperative X-rays by measuring the distance from the adductor tubercle to the joint line. All patients were operated on by two senior orthopedic arthroplasty surgeons. Either a cruciate retaining (CR) or a posterior stabilized (PS) Legion Total Knee Arthroplasty System (Smith & Nephew, Memphis, TN, USA) was used.

Ethical approval by the local institution review board was obtained prior to the study (EK 02/2023).

In all patients, either a medial parapatellar or midvastus approach was used based on the surgeon’s preference. No lateral parapatellar approach was used in our population. A conventional femoral cut-first technique was performed in all knees, based on the preoperative measured femoral correction angle, with external rotation aligned to the posterior condylar axis. The decision to use cruciate retaining TKA (CR-TKA) or posterior stabilized TKA (PS-TKA) depended on the intraoperative assessment of the posterior cruciate ligament (PCL) stability. In PS-TKA the PCL was removed; the implant consists of a central post and a cam mechanism, which compensates for the stability of the PCL. In CR-TKA the PCL remains; the implant relies on the PCL’s stability. The tibial cut was mechanically aligned and was performed with a conventional external alignment tower with a standard tibial slope of 3° of the cutting block. The tibial insert has a slope of 4°.

**Figure 1:**
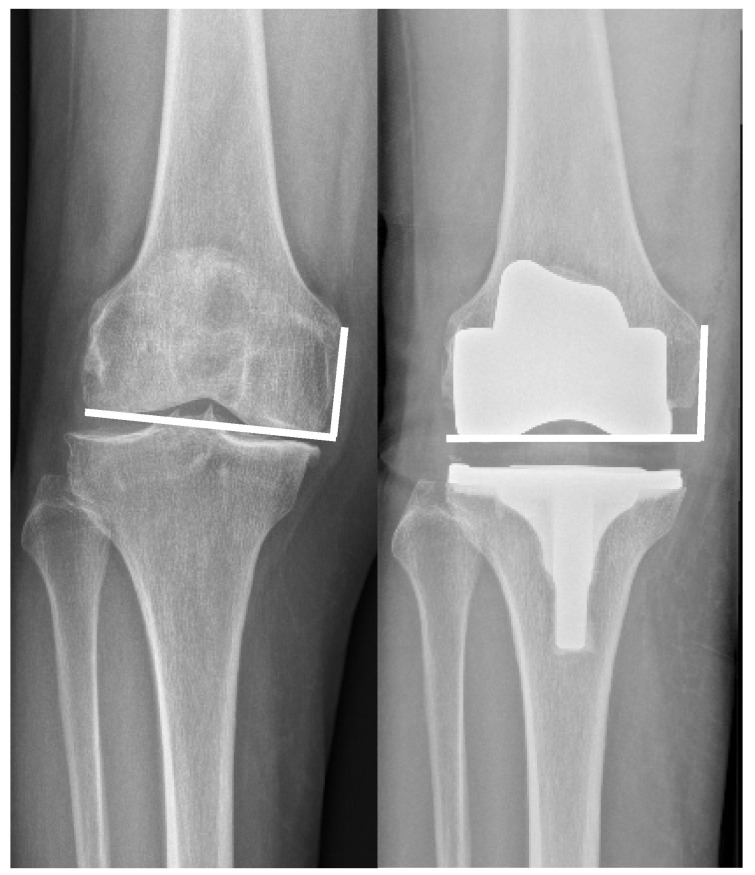
The joint line position was measured on pre- and postoperative anterior–posterior radiographs using the adductor tubercle to joint line distance as previously reported by Hofmann et al. [1].

All patients underwent pre- and postoperative standardized weight-bearing standing full-leg radiographs ranging from hip to ankle, anterior–posterior, as well as lateral knee radiographs. Varus and valgus deformities were measured on anterior–posterior full-leg hip-to-ankle radiographs according to Cook et al. [15]. Varus > 9.9° and Valgus > 9.9° were defined as severe deformities. The joint line was measured on pre- and postoperative anterior–posterior radiographs using the adductor tubercle (Figure 1) to joint line distance as previously reported by Hofmann et al. [1]. In accordance with prior reported data, the aim for joint line reconstruction was defined within four millimeters [1].

All measurements were performed by an orthopedic fellow at the senior author’s institution. The mediCAD^®^ software 7.0 (mediCAD Hectec GmbH, Altdorf/Landshut, Germany) was used for all measurements.

The data obtained was analyzed using the Statistical Package for Social Science (SPSS 24.0 version (IBM Corp., Armonk, NY, USA)). The data set was analyzed with the Shapiro–Wilk test for assessment of normality. The data set was found to have a normal distribution. Quantitative variables were expressed as mean ± SD. These were compared using an unpaired Student’s *t*-test while considering the difference in distribution among the two knee systems. Qualitative variables were expressed as frequencies and compared using the chi-square test and Fisher’s exact test whenever appropriate. A *p*-value <0.05 was considered statistically significant.

## 3. Results

A total of 67 (58.3%) women and 48 males (41.7%) with a mean age of 72.4 (±8.2 years, range 52 to 89 years) were included. Mean BMI was 28.5 kg/m^2^ (range 19.4–45.6 kg/m^2^). A total of 43 patients had a valgus deformity (range 0.1–16°), and 72 patients had a varus deformity (−0.4–−14.9°). A total of 36 patients (31.3%) underwent Cruciate Retaining (CR) TKA and 79 patients (68.7%) Posterior Stabilized (PS) TKA (Table 1).

There was no difference according to age between patients with varus (72.4 ± 8.1 years) and valgus deformity (72.4 ± 8.1 years; 72.3 ± 8.6 years; *p* = 0.924). Thirteen patients presented with a severe varus deformation of >−9.9° and 5 patients with a severe valgus of >9.9°. The age was also comparable between patients with a CR-TKA and a PS-TKA (71.2 ± 9.1 years; 72.9 ± 7.8 years; *p* = 0.31).

The preoperative (48.4 ± 6.4 mm) and postoperative (49.10 ± 6.3 mm) adductor tubercle to joint line distance in all knees slightly increased (0.6 ± 2.4 mm) and was statistically significantly different (*p* = 0.003). A total of 106 patients (92.2%) had the joint line restored within four millimeters. A total of 54 patients (47.0%) had joint line distalization within four mm, 33 patients (28.7%) had a joint line elevation within four millimeters, and 19 patients (16.5%) had an equal joint line. Seven patients (6.1%) had joint line distalization of more than four millimeters, and two patients (1.7%) had joint line elevation of more than four millimeters.

**Figure 2:**
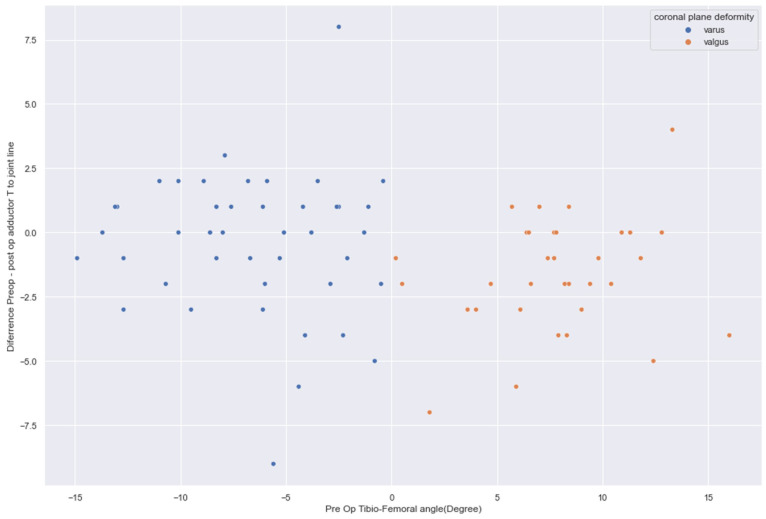
Differences from pre- to postoperative adductor tubercle to joint line in varus and valgus knees (mm).

**Figure 3:**
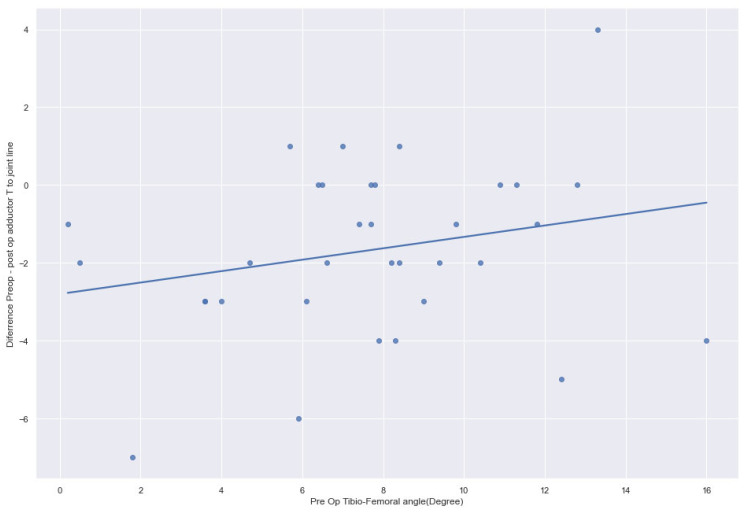
Pre-postoperative adductor tubercle to joint line elevation in valgus knees.

**Figure 4:**
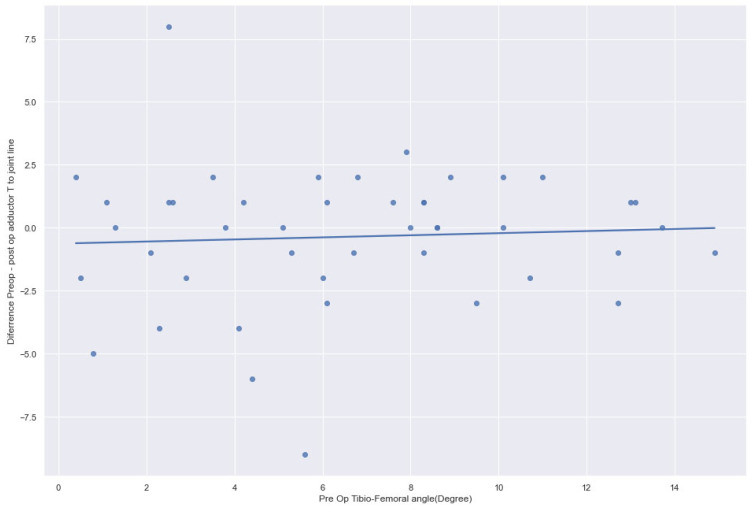
The joint line from pre- to postoperative adductor tubercle remained stable in varus knees.

In varus knees, the preoperative (48.56 ± 6.42 mm) and postoperative (48.86 ± 6.32 mm) joint lines remained statistically not significant (*p* = 0.31). In contrast, in valgus knees, the preoperative (47.9 ± 6.3 mm) and postoperative (49.3 ± 6.3 mm) joint lines were significantly greater (*p* < 0.0004) (Figure 2, Figure 3 and Figure 4). The preoperative adductor tubercle to joint line distance (varus preoperative 48.6 ± 6.42 mm, valgus preoperative 47.9 ± 6.3 mm, *p* = 0.585) and postoperative distance (varus postoperative 48.9 ± 6.3 mm, valgus postoperative 49.3 ± 6.3 mm, *p* = 0.717) in varus and valgus knees were not statistically significant. However, in valgus knees (1.4 ± 2.4 mm), the difference between the preoperative and postoperative adductor tubercle to joint line distance increased significantly more than in varus knees (0.3 ± 2.6 mm, *p* = 0.023). Preoperatively, valgus cases <9.9° presented with a joint line distance of 47.8 mm compared to 48.8 mm postoperatively (*p* = 0.001). Severe valgus cases >9.9° showed a distance of 48.8° preoperatively and 49.8° postoperatively (*p* = 0.342). The adductor to joint line distance in varus <−9.9° was preoperatively 48.3 mm and 48.6 mm postoperatively (*p* = 0.412). In severe cases, there was more than −9.9° varus deformity, the preoperative distance was 49.2 mm, and the postoperative distance was 49.5 mm (0.584).

A total of 34 valgus patients (79.1%) and 45 varus patients (62.5%) received a PS-TKA, which was not significantly different (*p* = 0.096). Patients with greater preoperative varus or valgus deformity (varus > 9.9°; valgus > 9.9°) did not show an increased degree of joint line change postoperatively (varus *p* = 0.71 vs. valgus *p* = 0.83). A total of 34 valgus patients (79.1%) and 45 varus patients (62.5%) received a PS-TKA, which was not significantly different (*p* = 0.096). Patients with greater preoperative varus or valgus deformity (varus > −9.9°; valgus > 9.9°) did not show an increased degree of joint line change postoperatively (varus *p* = 0.71 vs. valgus *p* = 0.83). Pearson correlation revealed no correlation in varus patients (−0.091) for joint line change. In valgus patients, the Pearson coefficient was 0.118.

## 4. Discussion

The current study compared joint line reconstruction in primary TKA and found that the adductor tubercle to joint line distance slightly increased radiologically from preoperative to postoperative measurements. Overall, 106 patients (92.2%) had the joint line restored within ± four millimeters. The study also examined whether varus or valgus deformity, as well as the use of CR or PS-TKA, influenced joint line reconstruction. In varus knees, the preoperative and postoperative joint lines were not statistically different; however, in valgus knees, a significant increase in the joint line was observed. By restoring the natural joint line, surgeons can significantly improve the longevity of TKA, minimize the risk of complications, and enhance functional recovery, especially in younger or more active patients. Additionally, restoring the natural joint line helps in possible revision surgery. Reasons for joint line elevation in valgus TKA, as shown in our study, include resecting more proximally to ensure adequate bone support or the need to compensate for soft tissue imbalance [16]. Chalmers et al. recommend the restoration of the joint line if possible; however, if joint line elevation is necessary, an intraoperative assessment of coronal laxity at 15° to 30° of knee flexion for mid-flexion instability should occur [17]. 

Hofmann et al. studied joint line changes in 89 revision TKAs using the adductor tubercle to joint line method. They used the +/− four millimeters as a cut-off method. A total of 59 patients had joint line restoration in this range, and they concluded clinical outcome was improved if the joint line was accurately improved [1].

Van Lieshout et al. [2] recommended that the joint line should not be elevated more than four mm. Babazadeh et al. and Yang et al. used two millimeters as a cut-off and found no correlation between International Knee Society Scores (IKSS) and joint lines if the postoperative joint line was maintained within two millimeters of the preoperative level. [18,19]. Yang et al. studied joint line variation in 50 knees operated on by navigation-assisted TKA and found that joint line variation in the range, from one mm to +five mm, was not associated with any adverse outcome [19]. To avoid mid-flexion instability, the reconstruction of the posterior offset within two mm is necessary according to Matziolois et al., although within a range between five mm proximalization and two mm distalization of the joint line, no influence on mid-flexion stability has been shown [7].

Ji et al. found no statistically significant difference in joint line changes between CR and PS knees in 55 primary TKA patients using the adductor tubercle to joint line method [20]. Snider et al.’s study used the fibular head as a landmark to measure the joint line and reported no statistically significant differences in the joint line changes between PS and CR designs within the same implant system [21]. Selvarajah and Hooper reported that using a CR prosthesis allows for a conservative tibial osteotomy, which can result in joint line elevation, with the goal of protecting the ligaments [22].

Schnurr et al. concluded that releasing or removing the posterior cruciate ligament (PCL), as performed in posterior-stabilized (PS) knee systems, increases the size of the flexion gap, thereby causing joint line elevation to balance the flexion-extension gap [23]. Additionally, Hino et al. found greater mid-flexion laxity in TKA with a PS prosthesis compared to a CR prosthesis, due to the increased flexion gaps resulting from cutting the PCL [24].

Cope et al. found no difference in polyethylene thickness comparing PCL-retaining and PCL-substituting TKA. The joint line position in their study preoperatively averaged 2.2 cm from the tibial tuberosity and postoperatively 2.4 cm in PCL-substituting and 2.5 cm in PCL-retaining TKA in their study, resulting in a change of two and three millimeters, respectively [25].

A comparison of the joint line in navigated versus non-navigated TKA in 493 primary TKA cases revealed a non-significant difference in joint line shift [26].

Robotic-assisted TKA has been shown to adequately restore joint line, patellar height, and condylar offset according to a study by Mayne et al. [27]. Popat et al. showed a more accurate restoration when comparing the conventional technique with robotic-assisted TKA [12].

Regarding joint line preservation, van Lieshout et al. recommend removing posterior osteophytes to counter a tight extension gap before recutting the distal femur, as posterior osteophytes tend to tighten up the posterior capsule and reduce the extension gap. In case of distal femoral bone loss, the distal femoral bone cut should be reduced [2].

Our study shows no difference in the preoperative and postoperative joint line in varus knees; however, in valgus knees, a significant increase in the joint line was observed. Constrained TKA for a valgus knee was shown to demonstrate significant joint line changes according to Pang et al., compared to unconstrained TKA [28]. Babazadeh et al. in their randomized controlled trial concluded a change in the joint line was associated with an alignment change, and an elevated postoperative joint line was linked to a larger preoperative malalignment [18].

The current study has the following limitations: Firstly, clinical data were not assessed; the practical impact remains unknown. Secondly, this study comprises a consecutive series of patients. No prior sample size calculation was performed, as the number of included patients reflects the available patient cohort during the study period. Consequently, the current study analyzes a limited number of patients and may be underpowered. Thirdly, all knees were performed using a conventional femoral cut first technique. Therefore, the accuracy of joint line reconstruction might be higher if computer-assisted TKA or patient-specific instrument-guided TKAs are used. The original anatomy of the patient might be influenced by degenerative changes and may not align with the natural axis. Patella height was not measured, possibly influencing the postoperative joint line. Fourthly, a selection bias might be present due to the fact we predominantly used PS-TKA, especially in valgus osteoarthritis.

## 5. Conclusions

The distance from the adductor tubercle to the joint line slightly increased in conventional total knee arthroplasty (TKA). However, in most patients, the joint line was restored within four millimeters. Surgeons should be aware of joint line elevation in valgus knees. In these patients, preoperative planning and measurement of native adductor tubercle to joint line distance might help to prevent joint line variation during surgery.

## Figures and Tables

**Table 1:** Demographics.

	PS-TKA	CR-TKA	*p*-Value
n	79	36	
Age	72.9 (52–88)	71.2 (55–89)	0.31
Male/Female	32/47	16/20	0.69
Varus/Valgus	45/34	27/9	0.096

## Data Availability

The original contributions presented in this study are included in the article. Further inquiries can be directed to the corresponding author.

## References

[1] Hofmann A.A., Kurtin S.M., Lyons S., Tanner A.M., Bolognesi M.P. (2006). Clinical and radiographic analysis of accurate restoration of the joint line in revision total knee arthroplasty. J. Arthroplast..

[2] van Lieshout W.A.M., Valkering K.P., Koenraadt K.L.M., van Etten-Jamaludin F.S., Kerkhoffs G., van Geenen R.C.I. (2019). The negative effect of joint line elevation after total knee arthroplasty on outcome. Knee Surg. Sports Traumatol. Arthrosc..

[3] Bellemans J. (2004). Restoring the joint line in revision TKA: Does it matter?. Knee.

[4] Stiehl J.B., Abbott B.D. (1995). Morphology of the transepicondylar axis and its application in primary and revision total knee arthroplasty. J. Arthroplast..

[5] Cross M.B., Nam D., Plaskos C., Sherman S.L., Lyman S., Pearle A.D., Mayman D.J. (2012). Recutting the distal femur to increase maximal knee extension during TKA causes coronal plane laxity in mid-flexion. Knee.

[6] Luyckx T., Vandenneucker H., Ing L.S., Vereecke E., Ing A.V., Victor J. (2018). Raising the Joint Line in TKA is Associated With Mid-flexion Laxity: A Study in Cadaver Knees. Clin. Orthop. Relat. Res..

[7] Matziolis G., Brodt S., Windisch C., Roehner E. (2017). Changes of posterior condylar offset results in midflexion instability in single-radius total knee arthroplasty. Arch. Orthop. Trauma Surg..

[8] Koshire S., Mohanty S.S., Keny S.A., Rai A.K., Rathod T.N., Kamble P. (2022). The influence of joint line restoration on functional outcome after primary total knee arthroplasty: A prospective study. J. Clin. Orthop. Trauma.

[9] König C., Sharenkov A., Matziolis G., Taylor W.R., Perka C., Duda G.N., Heller M.O. (2010). Joint line elevation in revision TKA leads to increased patellofemoral contact forces. J. Orthop. Res..

[10] Richards J.A., Williams M.D., Gupta N.A., Smith L.S., Malkani A.L. (2023). Neutral Mechanical Alignment Alters the Native Distal Femoral Joint Line: A Virtual Three-Dimensional Planning Total Knee Arthroplasty Study. J. Arthroplast..

[11] Niki Y., Kobayashi S., Nagura T., Udagawa K., Harato K. (2018). Joint Line Modification in Kinematically Aligned Total Knee Arthroplasty Improves Functional Activity but Not Patient Satisfaction. J. Arthroplast..

[12] Popat R., Albelooshi A., Mahapatra P., Bollars P., Ettinger M., Jennings S., Van den Berg J.L., Nathwani D. (2022). Improved joint line and posterior offset restoration in primary total knee replacement using a robotic-assisted surgical technique: An international multi-centre retrospective analysis of matched cohorts. PLoS ONE.

[13] Kowalczewski J.B., Labey L., Chevalier Y., Okon T., Innocenti B., Bellemans J. (2015). Does joint line elevation after revision knee arthroplasty affect tibio-femoral kinematics, contact pressure or collateral ligament lengths? An in vitro analysis. Arch. Med. Sci..

[14] Klemt C., Padmanabha A., Tirumala V., Smith E.J., Kwon Y.M. (2022). The Effect of Joint Line Elevation on In Vivo Knee Kinematics in Bicruciate Retaining Total Knee Arthroplasty. J. Knee Surg..

[15] Cooke T.D., Sled E.A., Scudamore R.A. (2007). Frontal plane knee alignment: A call for standardized measurement. J. Rheumatol..

[16] Del Gaizo D.J., Della Valle C.J. (2011). Instability in primary total knee arthroplasty. Orthopedics.

[17] Chalmers B.P., Elmasry S.S., Kahlenberg C.A., Mayman D.J., Wright T.M., Westrich G.H., Imhauser C.W., Sculco P.K., Cross M.B. (2021). Additional distal femoral resection increases mid-flexion coronal laxity in posterior-stabilized total knee arthroplasty with flexion contracture. Bone Jt. J..

[18] Babazadeh S., Dowsey M.M., Swan J.D., Stoney J.D., Choong P.F. (2011). Joint line position correlates with function after primary total knee replacement: A randomised controlled trial comparing conventional and computer-assisted surgery. J. Bone Jt. Surg. Br..

[19] Yang J.H., Seo J.G., Moon Y.W., Kim M.H. (2009). Joint line changes after navigation-assisted mobile-bearing TKA. Orthopedics.

[20] Ji S.J., Zhou Y.X., Jiang X., Cheng Z.Y., Wang G.Z., Ding H., Yang M.L., Zhu Z.L. (2015). Effect of Joint Line Elevation after Posterior-stabilized and Cruciate-retaining Total Knee Arthroplasty on Clinical Function and Kinematics. Chin. Med. J..

[21] Snider M.G., Macdonald S.J. (2009). The influence of the posterior cruciate ligament and component design on joint line position after primary total knee arthroplasty. J. Arthroplast..

[22] Selvarajah E., Hooper G. (2009). Restoration of the joint line in total knee arthroplasty. J. Arthroplast..

[23] Schnurr C., Eysel P., König D.P. (2012). Is the effect of a posterior cruciate ligament resection in total knee arthroplasty predictable?. Int. Orthop..

[24] Hino K., Ishimaru M., Iseki Y., Watanabe S., Onishi Y., Miura H. (2013). Mid-flexion laxity is greater after posterior-stabilised total knee replacement than with cruciate-retaining procedures: A computer navigation study. Bone Jt. J..

[25] Cope M.R., O’Brien B.S., Nanu A.M. (2002). The influence of the posterior cruciate ligament in the maintenance of joint line in primary total knee arthroplasty: A radiologic study. J. Arthroplast..

[26] Jawhar A., Shah V., Sohoni S., Scharf H.P. (2013). Joint line changes after primary total knee arthroplasty: Navigated versus non-navigated. Knee Surg. Sports Traumatol. Arthrosc..

[27] Mayne A.I., Rajgor H., Munasinghe C., Agrawal Y., Pagkalos J., Davis E.T., Sharma A.D. (2024). The ROSA robotic-arm system reliably restores joint line height, patella height and posterior condylar offset in total knee arthroplasty. Knee.

[28] Pang H.N., Yeo S.J., Chong H.C., Chin P.L., Chia S.L., Lo N.N. (2013). Joint line changes and outcomes in constrained versus unconstrained total knee arthroplasty for the type II valgus knee. Knee Surg. Sports Traumatol. Arthrosc..

